# The spatial pattern of *Populus euphratica* competition based on competitive exclusion theory

**DOI:** 10.3389/fpls.2024.1276489

**Published:** 2024-07-03

**Authors:** Yaxuan Liu, Yong Zeng, Peng Wang, Jia He, Pingping Li, Yuejia Liang

**Affiliations:** ^1^ College of Geographic Science and Tourism, Xinjiang Normal University, Urumqi, China; ^2^ Xinjiang Laboratory of Lake Environment and Resources in Arid Zone, Urumqi, China; ^3^ Xinjiang Institute of Ecology and Geography, Chinese Academy of Sciences, Urumqi, China

**Keywords:** *Populus euphratica*, competitive exclusion, competition, spatial pattern, regeneration

## Abstract

**Introduction:**

Population-level competition and spatial patterns may explain the role of competitive exclusion in communities, which is important for vegetation restoration and biodiversity conservation.

**Methods:**

We analyzed the competitive intensity, spatial patterns, and renewal of *Populus euphratica* Oliv. forests in the Tarim River Basin using the Hegyi competition index and spatial point pattern analysis in a completely random model with different habitats and different forest ages.

**Results:**

The greatest competitive distance for *P. euphratica* was 10 m, and the intensity of competition steadily decreased as the diameter increased. The intensity of intraspecific and interspecific competition in young, mature, and old *P. euphratica* forests was as follows: riverside habitat > transitional habitat > desert margin habitat. The Simpson index values for the three habitats decreased as follows: transitional > riverside > desert margin, and the Shannon-Wiener index and Pielou index values decreased as follows: riverside > transitional > desert margin. In the riverside habitat, the young *P. euphratica* forest experienced the greatest competitive intensity, the mature forest in the transitional habitat was the largest, and the forest in the desert margin habitat was the oldest. Competitive intensity was greatest in the young riverside *P. euphratica* forest, mature *P. euphratica* forest in the transitional habitat, and old forest in the desert margin. Riverside *P. euphratica* experienced strong competition from *Populus pruinosa*. Competitive exclusion caused *P. pruinosa* to disappear from the transitional and desert margin habitats. Young, mature, and old *P. euphratica* forests were randomly distributed along the riverside and in the transitional habitat, while mature and old *P. euphratica* forests were randomly distributed in the desert margin. *Populus pruinosa*, *Tamarix ramosissima*, and *Tamarix hispida* were mainly randomly distributed, and *T. ramosissima* and *T. hispida* were clustered at small scales. In the riverside habitat, young, mature, and old *P. euphratica* had no spatial correlation, and there was a significant negative correlation at small scales in the transitional habitat. The density of *P. euphratica* seedlings in the riverside habitat was greater than that in the transitional habitat, and greater competitive pressures on *P. euphratica* tree seedlings caused a lower renewal density.

**Conclusions:**

When planting *P. euphratica* forests, spacing greater than 10 m can effectively reduce stand competition and thus promote seedling regeneration.

## 1 Introduction

The ecological niche concept has interested ecologists since Grinnell proposed it in 1917, and the theory has been applied to study plant interspecific relationships, biodiversity, community structure, succession, and population evolution (Peng and Wang, 2016). The Russian biologist Gause (1934) defined the ecological niche as the position occupied by a specific species in the community, which included its habitat, food, and lifestyle (Whittaker et al., 1973; Li et al., 2006). The principle of competitive exclusion was proposed after experiments with different species of grasshoppers demonstrated that each species had a specific position in its community and that the differentiation of ecological niches was necessary for species to maintain coexistence in space and time (Hardin, 1960). Traditional theories of ecological niche differentiation were proposed at the beginning of the 20th century and included the role of habitat filtering (Keddy, 1992) and the principle of competitive exclusion (Gause, 1934). The principle of competitive exclusion primarily explains the role of the biological environment, suggesting that differences in ecological traits and life-history responses among species promote evolution and adaptation to finite sexual resources (Harpole and Tilman, 2006). In the same forest community, similarity between species will increase the intensity of competition and lead to competitive exclusion, thus weakening the similarity between species, generating niche differentiation, and ensuring the stability of coexisting species (May, 1973). Competition between species with the same resource utilization patterns prevents long-term stable coexistence (Vandermeer, 1972). Species that are not competitively dominant are eliminated because they cannot obtain resources for their growth, and, eventually, all species survive in suitable habitats, i.e., they obtain their ecological niches (Niu et al., 2009).

Over the long-term, organisms compete for living space, light, water, and food, thus forming relationships with each other (Zhuo, 2022). As an important interspecific relationship in terrestrial plant communities, competition influences the community structure and the evolutionary patterns of species. Darwin suggested that neighboring species with similar growth and development would form strong competitive relationships and that competitive exclusion would result in close relationships (Zhou, 2019); species with similar ecological niches cannot coexist in the same environment (Chai, 2016). Competitive relationships can influence the growth of individual trees (Luo et al, 2020), and they are also among the major forces influencing the structure and dynamics of forests, both of which are crucial to population renewal, succession, and the formation of spatial distribution patterns (Fraver et al, 2014; Zhang et al, 2016). Recent studies on *P. euphratica* have focused on population structure (Miao et al., 2020; Yusup et al., 2022), species diversity (Wang F. et al., 2020; Han and Wang, 2024), and spatial distribution (Yusup et al., 2022; He et al., 2023), but there have been few studies on intraspecific competition (He et al., 2023). Wang F. et al. (2020) studied the relationship between *P. euphratica* diversity and climate factors and concluded that *P. euphratica* diversity would expand from the center of China toward the northeast and northwest under future climate scenarios. Yusup et al. (2022) studied the population structure and spatial distribution of *P. euphratica* riparian forests along the Tarim River and reported that the age structure of the population increased in the upper reaches of the Tarim River, stabilized in the middle, and temporarily stabilized in the lower reaches; moreover, the decreasing depth of the groundwater and the soil water content increased the aggregation of *P. euphratica* along the Tarim River. He et al. (2023) studied variation in intraspecific competition and the spatial distribution of *P. euphratica* under different moisture gradients in the lower Tarim River and reported that with decreasing moisture, the competition index (CI) for *P. euphratica* stands decreased, and the spatial distribution gradually changed from an aggregated distribution to a random distribution. However, few studies on *P. euphratica* communities have applied the theory of competitive exclusion, and few have considered the common habitats of *P. euphratica*, such as the riverside, transitional, and desert margin habitats. Intraspecific and interspecific analyses based on the theory of competitive exclusion can therefore provide a theoretical basis for the conservation and restoration of poplar communities.

At present, mathematical models are the most effective method for studying competitive relationships (Miao, 2017). The CI can be classified into two categories: one is related to distance, and the other is independent of distance. Among the CI research methods related to distance, the Hegyi index proposed in 1974 is still widely used (Hegyi, 1974). Zhang et al. (2019) quantified individual CIs and their associations with tree diameter at breast height (DBH) using *Cunninghamia lanceolata* (Lamb.) with Hook as the test subject and Hegyi’s CI (distance-dependent). Luo et al. (2020) proposed an improved model based on the Hegyi CI to analyze whether the relationship between climate change and tree growth varied with tree-level competition. To improve predictions for *Cyclobalanopsis glauca* tree height, an improved CI was used by Long et al. (2020) to examine the effects of slope, gradient, and CI on the H-DBH relationship based on the Hegyi CI. Sun et al. (2022) calculated the CI components of trees in the four cardinal directions to indicate competitive pressure in all directions using the Hegyi CI. The Hegyi CI, which is characterized by high precision and flexibility, reflects the dominance of trees in a community and is suitable for studying community competition relationships in natural forests and in forests under various management measures (Zhang et al., 2012; Zhang Y. C., 2015).

The spatial pattern of a population is the distribution or configuration that characterizes all individuals in a given area, and it can be quantified to describe the distribution of the population (Velázquez et al., 2016). Biological properties, intraspecific and interspecific competition, environmental variation, and other factors combine to determine how populations are distributed in space (Harms et al., 2000). According to the degree and method of aggregation of populations, spatial distribution patterns are divided into three types: random distribution, uniform distribution, and aggregate distribution (Zhang et al., 2017). Different distribution patterns reveal different dynamic processes within the community, with aggregated distributions reflecting mutual reinforcement and similar resource requirements among species. Random distributions suggest that species compete for resources due to competitive exclusion (Liu, 2021). Interspecific associations, which are typically categorized into positive, negative, and no associations, refer to the spatial connectivity of various species. Spatial connectivity reflects the relationships formed via interaction between species within the community (Wen et al., 2022). A positive spatial correlation between two species indicates that their interaction is mutually beneficial and mutually reinforcing, a negative correlation indicates that two species are in a competitive and exclusive relationship, and no correlation indicates that the two species are independent of each other (Wang Y. F. et al., 2020). The study of spatial patterns of populations has long been a focus of ecology and has matured over the years. In a broad-leaved Korean pine forest in southeast Russia, Omelko et al. (2018) investigated the spatial distribution of tree species with various strategies, including saplings and adult trees. They found that species distribution patterns were significantly influenced by shade tolerance. Zhang et al. (2021) found that grazing altered the population multiplicity of species and the relationships among species in short-flowered needlegrass desert grasslands with different livestock loading rates. Chen et al. (2022) found that *Tamarix chinensis* in the muddy coastal zone of the Yellow River Delta had an aggregated distribution; aggregation intensity decreased with increasing spatial scale, and the aggregated distribution shifted to a random distribution with increasing spatial scale. Liu et al. (2023) investigated plants in the Nanling Mountains and found that the dominant *Machilus chinensis* had three spatial distribution states (aggregated, random, and uniform), while *Pinus massoniana* was randomly distributed, and the remaining species were aggregated at a small scale and randomly distributed at a large scale.

Competitive exclusion has an important impact on natural regeneration processes. Interspecific competition in resource-limited environments can reduce or cause the disappearance of populations of certain species, thus affecting their natural regeneration capacity (Gao, 2020). Competitive exclusion also affects the population size and distribution of species (Liu Y., 2022). Plant succession and maintenance, plant dynamics, forest resource regeneration and ecosystem recovery (Khaine et al., 2018; Shen and Nelson, 2018), and the sustainable growth of trees are influenced by natural renewal (Wand and Hao, 2012). Natural renewal plays an extremely important role in maintaining biodiversity and ecosystem productivity (Sharma et al., 2018; Mores et al., 2020; Zhao, 2021; Sun et al., 2024). The growth and development of tree seedlings is crucial to the process of regeneration (Muller-Landau et al., 2008). Moisture is an important factor influencing the distribution and regeneration of *P. euphratica* forests (Han et al., 2013). In summer, alpine ice and snow melt occurs in the upper reaches of the Tarim River and flows into the river channel, causing seasonal flooding in poplar forests on both sides of the river (Wang X. Y. et al., 2020). *Populus euphratica* trees are resistant to periodic flooding, which allows them to grow in riverside areas where water conditions are dynamic (Frymark-Szymkowiak and Kieliszewska-Rokicka, 2023). The flood plain provides suitable habitat conditions (low salt, high water, and clay soil) for the natural germination of riparian plant seeds and is ideal for *P. euphratica* (Li J. et al., 2013; Lei et al., 2020), and groundwater in the floodplain habitat fluctuates frequently. Good and bad habitats for *P. euphratica* forests in the Tarim River Basin are determined by groundwater depth, which directly affects the growth and density of *P. euphratica* (Li et al., 2003), as well as the growth and regeneration of young forests (He, 2022). Although *P. euphratica* can tolerate extreme drought, it is highly dependent on groundwater and sensitive to groundwater changes. The degree of seedling regeneration determines population renewal and affects the distribution of individuals and the structural composition of the population (Shu et al., 2019). However, during seedling growth, plants are extremely sensitive to changes in the environment (Svenning et al., 2008), especially the effects of competitive action caused by biological factors (Blank, 2010). Therefore, research on the competitive ability of forest seedlings is necessary to understand forest regeneration and provide a foundation for plantation forest creation.

In this study, we investigated competition and spatial patterns in *P. euphratica* communities in the Tarim River Basin based on the theory of competitive exclusion. Our main objectives were to: (i) determine whether the competitive range, competitive intensity, species diversity, spatial pattern, and seedling regeneration in poplar communities in different habitats had changed and (ii) determine whether competitive intensity and spatial patterns of *P. euphratica* and *Populus pruinosa* in riversides, transitional habitats, and desert margins conformed to the principle of competition exclusion. Addressing these objectives will improve our understanding of the mechanism of community building in the Tarim River and support the conservation and restoration of *P. euphratica*.

## 2 Materials and methods

### 2.1 Study area

The Tarim River Basin is the largest inland river basin in China. It is situated in the southern section of the Xinjiang Uygur Autonomous Region (71°39′-93°45′E, 34°20′-43°39′N) and covers a total area of 1.028 × 10^6^ km^2^. The middle reaches of the Tarim River occur from Imbaza to Chala, with a length of 398 km. Abundant water enters the mainstream of the Tarim River from melting mountain ice and snow (Liu et al., 2019); the water volume varies greatly from year to year, and the riverbank is sufficient. The study area is located at the northern edge of the Taklamakan Desert, where precipitation is scarce, and evaporation is very strong. The average annual precipitation is approximately 550 mm and evaporation is as high as 1125–1600 mm; the climate is hot and dry (Gou et al., 2017). Due to harsh environmental conditions, vegetation is distributed in bands along the river. Vegetation types are characterized by a general top-to-bottom transition from high-cover *P. euphratica* forests, *T. chinensis* shrubs, and *Halogeton glomeratus* belts, to medium-cover *P. euphratica* forests and *T. chinensis* shrubs, to low-cover sparse *T. chinensis* shrubs (Li M. Y. et al., 2021).

### 2.2 Research methods

#### 2.2.1 Data collection

**Figure 1:**
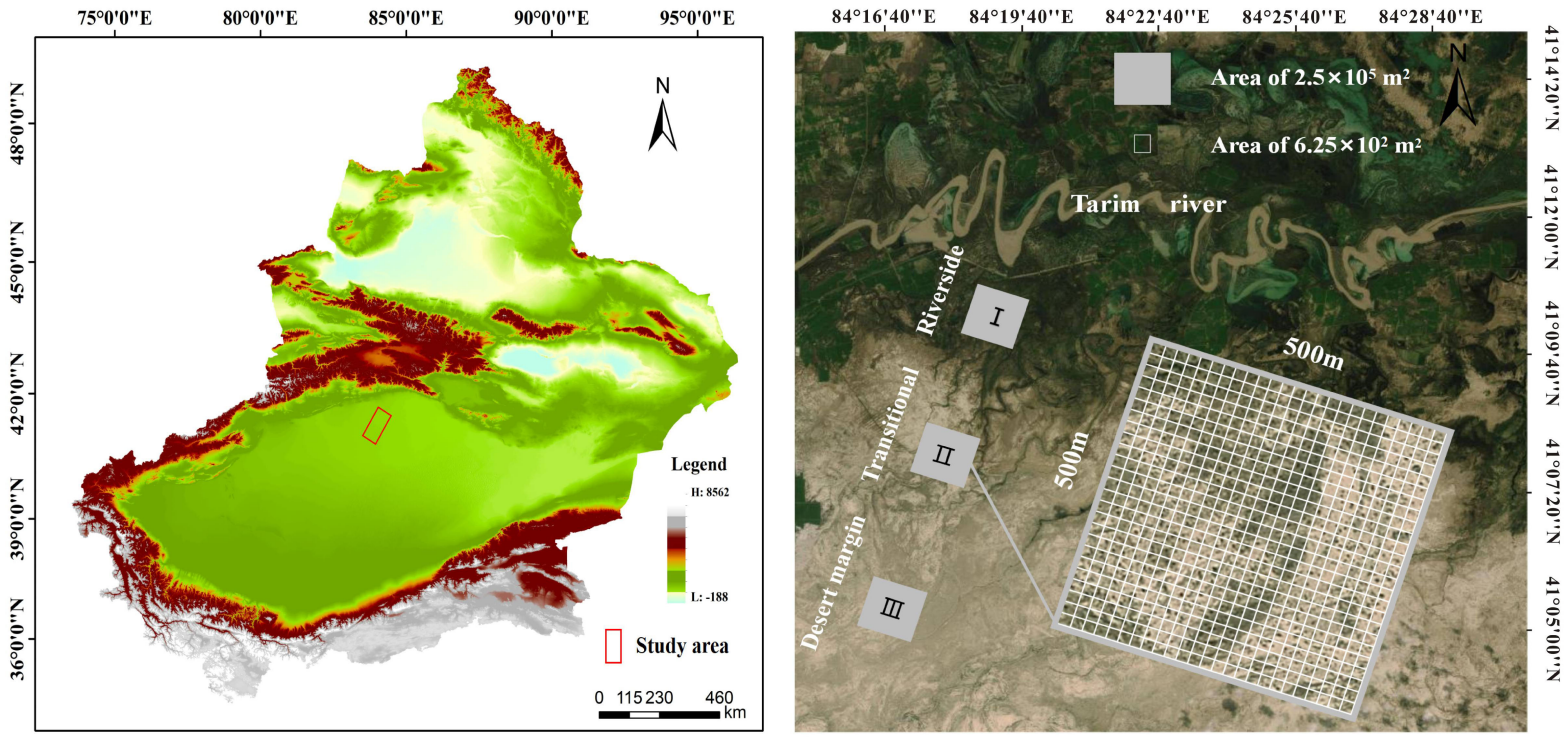
An overview map of the Tarim River Basin research area.

**Figure 2:**
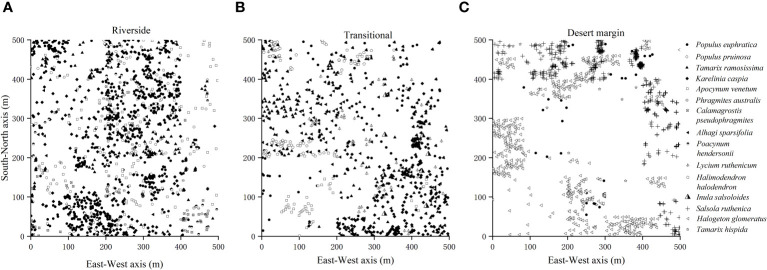
Survey of species distribution in the riverside **(A)**, transitional **(B)**, and desert margin habitats **(C)**.

Representative natural *P. euphratica* forests were selected for field survey and sampling along the riverside habitat, transitional habitat, and desert margin habitat of the Tarim River. One 500 m × 500 m community plot was set up for each habitat, with geographic coordinates as follows: 41°11′7.79″N, 84°19′9.37″E; 41°7′20.27″N, 84°17′45.73″E; and 41°4′9.33″N, 84°14′56.40″E. Each 500 m × 500 m plot was divided into 400 25 m × 25 m quadrats using the adjacent grid method, making 1200 quadrats in total (**Figure 1**). Species, tree height, DBH, status (alive or dead), and relative spatial coordinates (perpendicular distance from the XY axis) were recorded within each plot. The spatial coordinate data for the forest trees in the plots in **Figure 2** were investigated using a *P. euphratica* object tree as the center of the circle in the quadrat, and the distances between all competing trees and the object tree in the sample circle were measured. In each sample plot, NMR (Vista Clara, Inc., developed by GMR) and ground penetrating radar (Italy RIS-2K) were used to identify underground water that was deeply buried (Zeng et al., 2020). Soil from 0–20 cm was collected from five points in each plot, mixed, air-dried, and passed through a 2 mm sieve. Soil conductivity was evaluated by leaching air-dried soil in a leaching solution with distilled water (5:1 water:soil ratio) at 25°C. Total salt was determined by the dry slag method. The soil moisture content was determined indoors using the aluminum box drying method. Soil samples were sieved with a 0.25 mm sieve, and soil organic matter was evaluated using an external heating method with potassium dichromate. Each plot was localized using a Global Position System device.

#### 2.2.2 Identifying the optimal competitive range of neighbors

We randomly selected 176, 41, and 7 P*. euphratica* object trees in the riverside, transitional, and desert margin habitats, respectively, and set up a sample circle with each *P. euphratica* tree at the center; 1–20 m (with a grade difference of 1 m) was used as the radius of the sample circle. Within each sample circle, the average CI of the competing tree was calculated, and the relationship between the radius of each circle and the corresponding average CI was fitted using regression.

#### 2.2.3 Standards for dividing *P. euphratica* into different developmental stages

Age structure is an important indicator of population dynamics. Size order is commonly used for single plants, and diameter order is commonly used for tree species instead of age structure. According to domestic classification standards and its life history characteristics, *P. euphratica* was divided into young forest (0 cm< DBH< 16 cm), mature forest (20 cm ≤ DBH< 40 cm) and old forest (DBH ≥ 40 cm) (Wang et al., 2017).

### 2.3 Data analysis

#### 2.3.1 Competition index model

The intraspecific and interspecific CIs for *P. euphratica* were calculated using Hegyi (1974) single-tree competition model, as shown in Equations 1–2.


(1) $$CI_{i}=\sum_{j=1}^{n}D_{j}/\left(D_{i}\times L_{ij}\right)$$

(2) $$CI=\sum_{i=1}^{N}CI_{i}$$

where *CI*_i_ is the competition index of the *i*th object tree; n is the number of competing trees around the *i*th object tree; and *D_i_* and *D_j_* are the diameter at breast height (cm) of target tree *i* and competing tree *j*, respectively. *L*_ij_ is the separation in meters between object tree *i* and competing tree *j*; *CI* is the population competition index. The population’s total number of object trees is *N*. The level of competition increases with the *CI* value.

#### 2.3.2 Competition intensity and diameter correlation

Competition intensity and *P. euphratica* DBH were used for regression analysis. The independent variable was *P. euphratica* DBH, and the dependent variable was the CI. The power function regression fitting relationship was shown in Equation 3 (Xiang et al., 2015):


(3) $$CI=AD^{-B}$$

where *CI* is the competition index, *D* is the object tree’s DBH, and *A* and *B* are model parameters.

#### 2.3.3 Point pattern analysis

The Ripley (1977) function, which accounts for the number of individual plants in a circle of specific radius length *r* within the sample square is the most frequently used function in point pattern analysis. Its expression was shown in Equation 4:


(4) $$K\left(r\right)=\frac{S}{n^{2}}\sum_{i=1}^{n}\sum_{j=1}^{n}\frac{I_{r}\left(u_{ij}\right)}{w_{ij}}\left(i\neq j\right)$$

where *r* is the spatial scale in m, *S* is the sample plot size in m^2^, n is the total number of individuals in the sample plot, and *u_ij_* is the distance between two individuals *i* and *j*. *I_r_* (*u_ij_*) represents the characteristic function. When *u_ij_* ≤ r, *I_r_* (*u_ij_*) = 1 and if *u_ij_* > r, *I_r_* (*u_ij_*) = 0. *W_ij_* is the ratio of the arc length of the sector in region *A* of the circle with *i* as the center and *u_ij_* as the radius, to eliminate the boundary effect.

At the same time, a *g*(*r*) function that overcomes the cumulative effect of this function was introduced (Li X. et al., 2020); *g*(*r*) includes the univariate correlation analysis *g*_11_(*r*) function and the bivariate analysis *g*_12_(*r*) function (Cui et al., 2021). The *g*_11_(*r*) function, which reflects the probability of a species occurring within a given circle width with any point as the circle’s center and a specific distance (scale) as the radius, can be used to study the spatial distribution pattern of a particular population. The aggregation intensity is represented by the value of *g*_11_(*r*), as shown in Equation 5:


(5) $$g_{11}\left(r\right)=\frac{1}{2\pi r}\times \frac{dK(r)}{d(r)}$$

where d*K*(*r*) is the derivative of the function *K*(*r*) and *d*(*r*) is the derivative of the radius *r*. When *g*_11_(*r*) = 1, the population is said to be randomly distributed. When *g*_11_(*r*) > 1, the population is clustered. When *g*_11_(*r*)< 1, the population is uniformly distributed. To examine the spatial correlation between two species, the bivariate pairwise correlation function *g*_12_(*r*) was used, as shown in Equation 6. The following expression represents the likelihood that another species will exist within a circle of a given width that is centered on a member of one species and has a given distance (scale) as its radius:


(6) $$g_{12}\left(r\right)=\frac{1}{2\pi r}\times \frac{dK_{12}\left(r\right)}{d_{12}\left(r\right)}$$

where d*K*_12_(*r*) is the derivative of the function *K*(*r*) and *d*_12_(*r*) is the derivative of the radius *r*. When *g*_12_(*r*) = 1, there is no association between the two species; when *g*_12_(*r*) > 1, the relationship between the two species is positive; and when *g*_12_(*r*)< 1, the relationship between the two species is negative.

#### 2.3.4 Analysis of population renewal

Seedlings within the radius of the 5 m and 10 m sample circles were counted, and the renewal density of the sample circle area was computed as follows: renewal density = number of seedlings/circular area (Liu et al., 2018). Regression analysis was conducted using the competition intensity of *P. euphratica* and its renewal density within the radius of the sample circle. The CI was the dependent variable, and the renewal density inside the sample circle’s radius was the independent variable. The power function regression fitting relationship was shown in Equation 7:


(7) $$CI=AR^{-B}$$

where *CI* denotes the competitiveness index, *R* is the renewal density within the sample circle’s radius, and *A* and *B* denote the model parameters.

### 2.4 Statistical analysis

A zero model can help determine the spatial pattern type of a population. We adopted the complete spatial randomness (CSR), a mean Poisson process, as the zero model for testing (Wiegand et al., 2007). The upper and lower wrapped trajectory lines served as a significance test to determine whether the results deviated from the random state, and the maximum and minimum values of the simulations were used to generate the upper and lower wrapped trajectory lines, respectively. Ninety-nine Monte Carlo stochastic simulations were used to construct 95% confidence intervals for the corresponding point pattern functions.

## 3 Results

### 3.1 Overview of *P. euphratica* object trees and competing trees

The population profiles and environmental factors for *P. euphratica* in different habitats are shown in **Table 1** and **Table 2**. In this study, 224 P*. euphratica* object trees and 680 P*. euphratica* competing trees were selected (**Table 1**). The riverside habitat contained the largest proportion of young forest, with 147 young forest object trees, which accounted for 82.12% of the total, and 448 young forest competing trees, which accounted for 83.43%. The mature forest in the transitional habitat contained 26 mature forest object trees, which accounted for 63.41% of the total, and 78 mature forest competing trees, which accounted for 63.93%. The proportion of old forest was largest in the desert margin habitat, with 5 old forest target trees that accounted for 71.43% of the total and 14 old forest competing trees that accounted for 66.67%.

**Table 1:** Overview of *Populus euphratica* object trees and competition trees.

		Object tree			Competitive tree		
Habitat	Forest age	Number	Proportion (%)	Average DBH(cm)	Number	Proportion (%)	Average DBH(cm)
Habitat	Forest age	Number	Proportion (%)	Average DBH(**cm)**	Number	Proportion (%)	Average DBH(**cm)**
Riverside	Young forest (0 cm<DBH<16 cm)	147	82.12%	4.30	448	83.43%	4.44
	Mature forest (20 cm≤DBH<40 cm)	26	14.53%	30.81	84	15.64%	27.47
	Old forest(DBH≥40 cm)	3	1.68%	46.00	5	0.93%	45.00
Transitional	Young forest (0 cm<DBH<16 cm)	13	31.71%	6.99	40	32.79%	6.67
	Mature forest (20 cm≤DBH<40 cm)	26	63.41%	29.91	78	63.93%	29.43
	Old forest(DBH≥40 cm)	2	4.88%	49.00	4	3.28%	45.50
Desert margin	Young forest (0 cm<DBH<16 cm)	0	0	0	0	0	0
	Mature forest (20 cm≤DBH<40 cm)	2	28.57%	25.00	7	33.33%	25.00
	Old forest(DBH≥40 cm)	5	71.43%	44.00	14	66.67%	43.00

**Table 2:** Environmental factors in the riverside, transitional and desert margin habitats.

	Riverside	Transitional	Desert margin
River distance (km)	0.58	8.04	14.77
PH	8.69 ± 0.42a	7.94 ± 0.25b	7.84 ± 0.05b
**Conductivity**	10.12 ± 1.38a	4.40 ± 0.89b	2.99 ± 0.76b
**Total salt**	46.49 ± 8.31a	17.65 ± 2.41b	11.93 ± 2.52b
**Groundwater Depth (m)**	2.33 ± 0.10a	4.67 ± 0.21b	15.73 ± 1.12c
Soil moisture content	21.43 ± 3.41a	10.10 ± 9.54b	1.59 ± 1.19b
**Soil organic matter**	5.67 ± 1.10a	2.96 ± 0.46b	1.02 ± 0.55c
**Soil organic carbon**	7.97 ± 2.54a	4.26 ± 1.15b	1.99 ± 0.97b
**Whether affected by flood overflows**	Yes	No	No

In the study area, 13 species of competing trees in addition to *P. euphratica* were investigated (**Table 3**). There were 8 species of competing trees in the riverside habitat, including *P. pruinosa*, *Tamarix ramosissima*, *Karelinia caspia*, *Apocynum venetum*, and *Phragmites australis*. There were 7 species of competing trees in the transitional habitat, including *T. ramosissima*, *K. caspia*, *A. venetum*, and *Alhagi sparsifolia*. There were 3 species of competing trees in the desert margin habitat, including *Tamarix hispida*, *H. glomeratus*, and *Salsola ruthenica.*

**Table 3:** Competitive tree species composition and competitive intensity.

Habitat	Species	Average DBH/ Basal Diameter (cm)	Number	Proportion (%)	Total Competition Index	Young forest	Mature forest	Old forest
Riverside	Populus euphratica*	8.422	537	39.51%	289.908	274.452	13.445	2.011
	*Populus pruinosa*	18.859	118	8.68%	90.714	90.240	0.411	0.063
	*Tamarix ramosissima*	6.478	232	17.07%	18.104	17.945	0.137	0.022
	*Karelinia caspia*	–	165	12.14%	–	–	–	–
	*Apocynum venetum*	–	119	8.76%	–	–	–	–
	*Phragmites australis*	–	81	5.96%	–	–	–	–
	*Calamagrostis pseudophragmites*	–	51	3.75%	–	–	–	–
	*Alhagi sparsifolia*	–	28	2.06%	–	–	–	–
	*Poacynum hendersonii*	–	28	2.06%	–	–	–	–
	Total	–	1359	100%	398.726	382.637	13.993	2.096
Transitional	Populus euphratica*	15.477	122	12.42%	12.943	1.941	10.490	0.512
	*Tamarix ramosissima*	6.506	241	24.54%	1.939	1.842	0.084	0.013
	*Lycium ruthenicum*	0.684	73	7.43%	0.154	0.131	0.023	–
	*Halimodendron halodendron*	0.755	92	9.37%	0.098	0.082	0.016	–
	*Alhagi sparsifolia*	–	239	24.34%	–	–	–	–
	*Karelinia caspia*	–	163	16.60%	–	–	–	–
	*Apocynum venetum*	–	44	4.48%	–	–	–	–
	*Inula salsoloides*	–	8	0.81%	–	–	–	–
	Total	–	982	100%	15.134	3.996	10.613	0.525
Desert margin	Populus euphratica*	16.810	21	1.76%	0.401	0	0.134	0.267
	*Tamarix hispida*	15.556	18	1.51%	0.140	0.102	0.038	–
	*Halogeton glomeratus*	–	567	47.61%	–	–	–	–
	*Salsola ruthenica*	–	585	49.12%	–	–	–	–
	Total	–	1191	100%	0.541	0.102	0.172	0.267

* Represents intraspecific competition, the rest represents interspecific competition.

### 3.2 *P. euphratica* competition intensity

#### 3.2.1 Determination of the optimal competitive range

**Figure 3:**
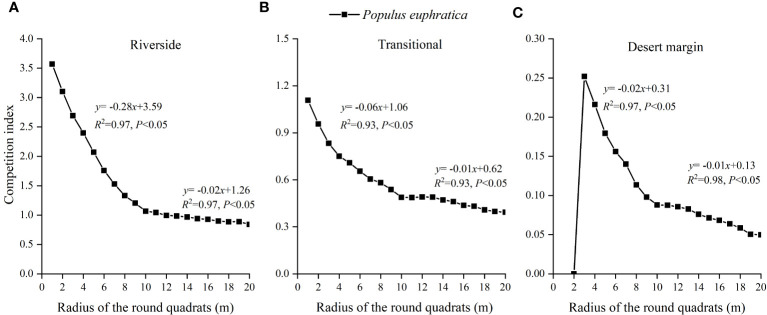
Relationship between the radius of *P. euphratica* sample circle and the change of competition index in the riverside **(A)**, transitional **(B)**, and desert margin habitats **(C)**.

**Figure 3** shows that the average CI gradually decreased as the distance between competing trees increased, with a clear inflection point at 10 m. When the radius of the sample circle was greater than 10 m, the CI changed slowly. At less than 10 m, the CI changed quickly and showed an obvious downward trend. The segmented fitting findings of the association between various sample circle radii and competition intensity also showed that the two fitted equations reached a significant level and that the maximum *R* ^2^ occurred for the sample circle radii in the range of 1–10 m and 10–20 m. Therefore, the distance between the object tree and the competing tree was 10 m, which was the most suitable competitive range for studying the competitive intensity of *P. euphratica*.

#### 3.2.2 Intraspecific and interspecific competition intensity for *P. euphratica*

**Figure 4:**
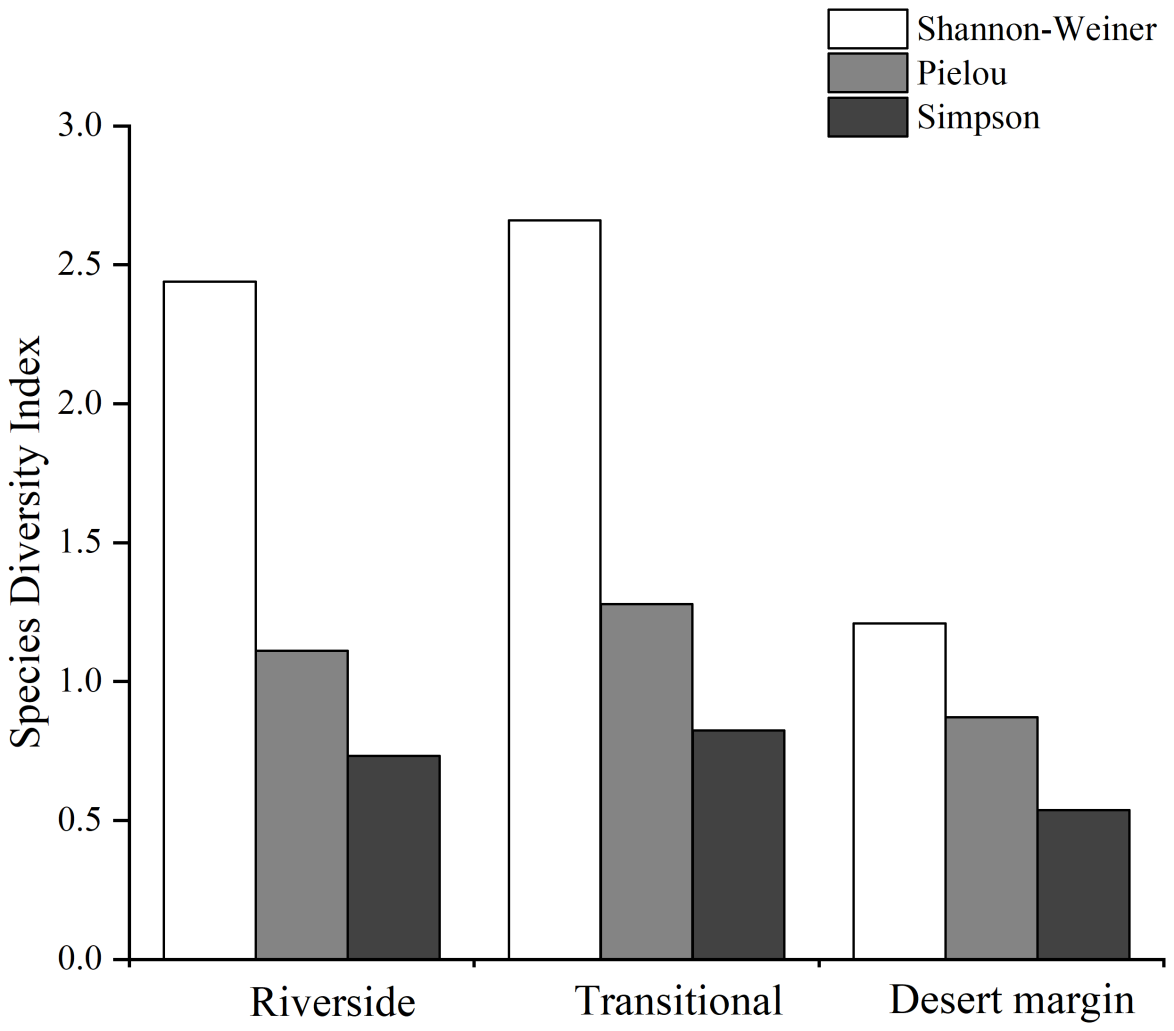
Species diversity along the riverside, transitional, and desert margin habitats.

The total intensity of intraspecific competition was 303.252, which accounted for 73.11% of the total competition intensity, and the total intensity of interspecific competition was 111.55, which accounted for 26.89% of the total competition intensity, indicating that *P. euphratica* experienced more intraspecific competition (**Table 3**). The Simpson index values for the three habitats decreased from the transitional habitat to the riverside habitat to the desert margin habitat, and the Shannon-Wiener index and Pielou index values decreased from the riverside habitat to the transitional habitat to the desert margin habitat (**Figure 4**). The intraspecific competition intensity of *P. euphratica* along the riverside decreased with increasing age of the forest. The competition intensity of the young forest was 274.452, that of the mature forest was 13.445, and that of the old forest was 2.011, indicating that intraspecific *P. euphratica* competition along the riverside mainly occurred in the young forest. In the transitional habitat, intraspecific *P. euphratica* competition mainly occurred in the mature forest, and the intensity of intraspecific competition decreased from the mature forest to the young forest to the old forest. In the desert margin habitat, intraspecific *P. euphratica* competition mainly occurred in the old forest, and the intensity of intraspecific competition was greater in the old forest than in the mature forest.

Among the companion species of *P. euphratica*, the greatest intensity of competition in the riverside habitat was 90.714 for *P. pruinosa*, followed by 18.104 for *T. ramosissima*. The greatest competitive intensity in the transitional habitat was 1.939 for *T. ramosissima*, and the lowest competitive intensity was 0.098 for *Halimodendron halodendron*. The greatest competitive intensity in the desert margin habitat 0.140 for *T. hispida*.

#### 3.2.3 Relationship between the intensity of competition in *P. euphratica* and the diameter of the object tree

**Figure 5:**
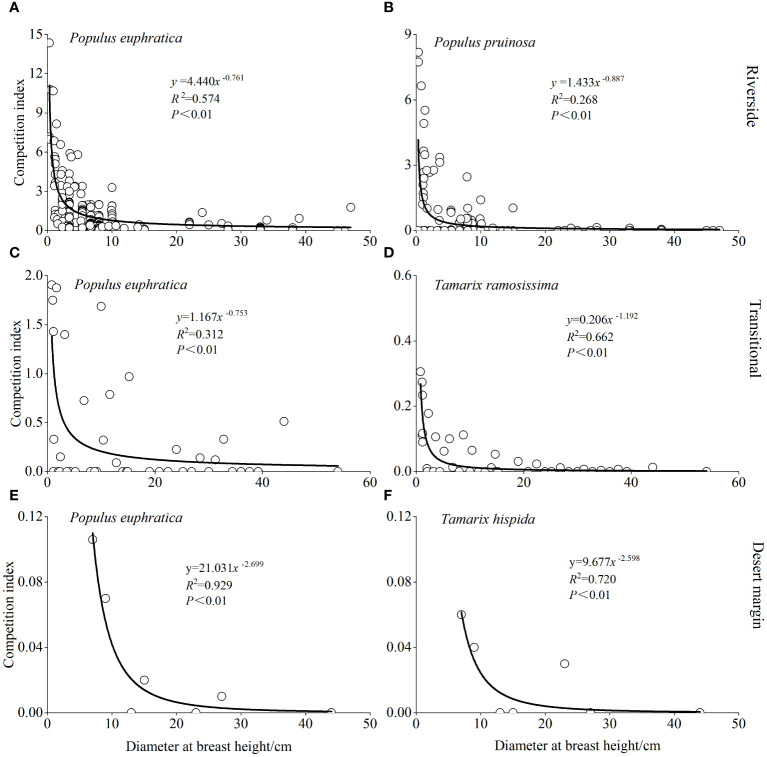
Model predictions of intraspecific competitive intensity and object trees diameter at breast height for *P. euphratica* in the riverside **(A)**, transitional **(C)**, and desert margin habitats **(E)**. Model predictions of interspecific competitive intensity and object trees diameter at breast height in *P. euphratica* and *P. pruinosa* in the riverside habitat **(B)**. Model predictions of interspecific competitive intensity and object trees diameter at breast height in *P. euphratica* and *T. ramosissima* in the transitional habitat **(D)**. Model predictions of interspecific competitive intensity and object trees diameter at breast height in *P. euphratica* and *T. hispida* in the desert margin habitat **(F)**.

The relationship between DBH and the intraspecific *P. euphratica* CI in the riverside, transitional, and desert margin habitats is represented by the power functions *CI* = 4.440*D* ^-0.761^ (*R* ^2^ = 0.574, *P* < 0.001), *CI* = 1.167*D* ^-0.753^ (*R* ^2^ = 0.312, *P* < 0.001), and *CI* = 21.031*D* ^-2.699^ (*R* ^2^ = 0.929, *P* < 0.001), respectively (**Figure 5**). The relationship between the DBH of *P. euphratica* object trees and the interspecific CI for *P. euphratica* and *P. pruinosa* in the riverside habitat was *CI* = 1.433*D* ^-0.887^ (*R* ^2^ = 0.268, *P* < 0.001). The relationship between the DBH of *P. euphratica* object trees and the interspecific CI for *P. euphratica* and *T. ramosissima* in the transitional habitat was *CI* = 0.206*D* ^-1.192^ (*R* ^2^ = 0.662, *P* < 0.001). The relationship between the DBH of *P. euphratica* object trees and the interspecific CI for *P. euphratica* and *T. hispida* in the desert margin habitat was *CI* = 9.677*D* ^-2.598^ (*R* ^2^ = 0.720, *P* < 0.001). Intraspecific and interspecific competition intensities for *P. euphratica* decreased as DBH increased. When DBH was less than 30 cm, the competition intensity for *P. euphratica* increased significantly. When DBH was greater than 30 cm, *P. euphratica* competition was weak, the decline was small, and it was maintained at a low level.

### 3.3 Spatial pattern analysis for *P. euphratica*

#### 3.3.1 Spatial distribution of *P. euphratica* in forests of different ages

**Figure 6:**
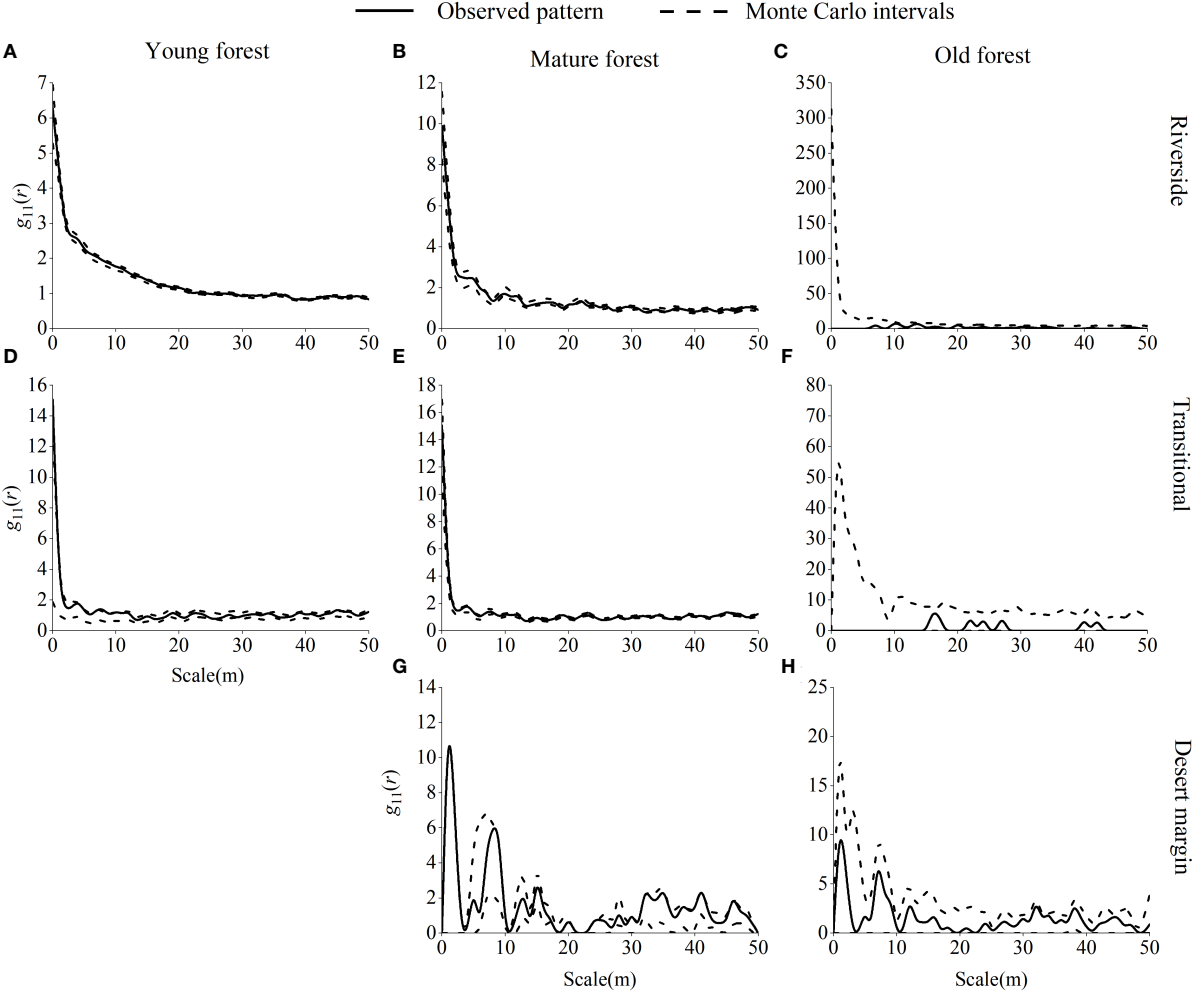
Spatial distribution pattern of *P. euphratica* in different age forests in the riverside **(A–C)**, transitional **(D–F)**, and desert margin habitats **(G, H)**. The spatial distribution of a population is represented by *g*_11_(*r*).

Young *P. euphratica* forests were randomly distributed along the riverside, were mainly randomly distributed in the transitional habitat, and were aggregated at 8 m, 10 m, and 45 m scales (**Figure 6**). Mature *P. euphratica* forests were randomly distributed in the riverside and desert margin habitats and were mainly randomly distributed in the transitional habitat, with a uniform distribution at 26 m and 36 m scales. *P. euphratica* old-growth forests were randomly distributed in the riverside, transitional, and desert margin habitats.

**Figure 7:**
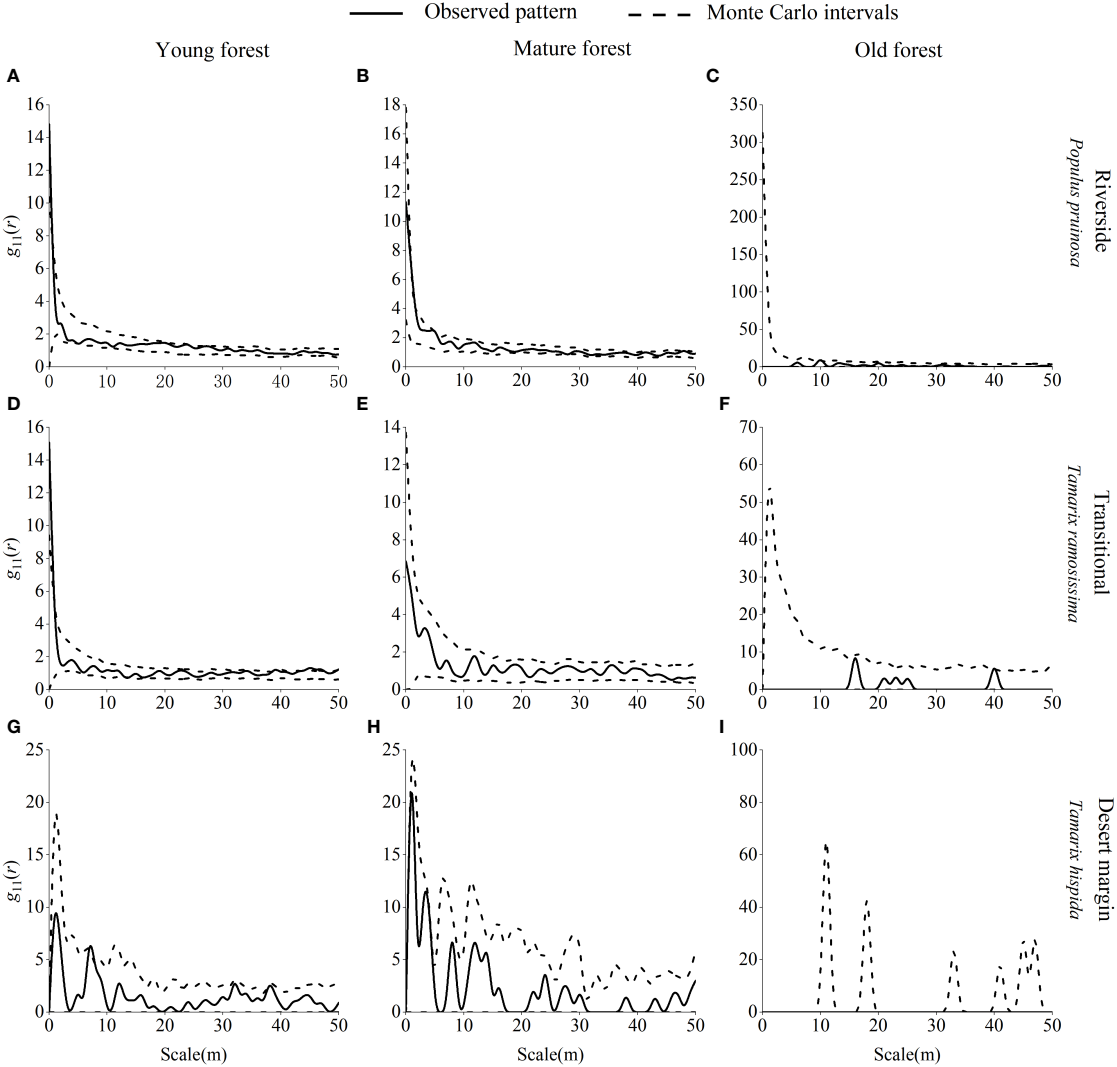
Spatial distribution pattern of *P. pruinosa*, *T. ramosissima* and *T. hispida* in different age forests. Spatial distribution pattern of *P. pruinosa* in the riverside **(A–C)**, *T. ramosissima* in the transitional **(D–F)**, and *T. hispida* in the desert margin habitat **(G–I)**.

Young *P. pruinosa* stands were mainly randomly distributed along the riverside and clustered at 0 m, 20 m, 24 m, 27 m, and 31 m scales (**Figure 7**). Young *T. ramosissima* stands were predominantly randomly distributed in the transitional habitat, with clustered distributions at 0 m, 34 m, 39 m, 41 m, 43–47 m, and 50 m scales in the transitional habitat and uniform distributions at 6 m and 16 m scales. Young stands of *T. hispida* were clustered in the desert margin at 7 m, 32 m, and 38 m scales. Mature stands of *P. pruinosa* were predominantly randomly distributed along the riverside, with clustering only at the 1 m and 5 m scales. Mature *T. ramosissima* and *T. hispida* forests were randomly distributed in the transitional and desert margin habitats. Old growth *P. pruinosa* forests were mainly randomly distributed along the riverside, with clustering only at the 10 m scale. Old growth *T. ramosissima* and *T. hispida* forests were predominantly randomly distributed in both the transitional and desert margin habitats, and *T. ramosissima* old-growth forests were clustered only at the 44 m scale in the transitional habitat.

#### 3.3.2 *P. euphratica* spatial correlation in forests of different ages

**Figure 8:**
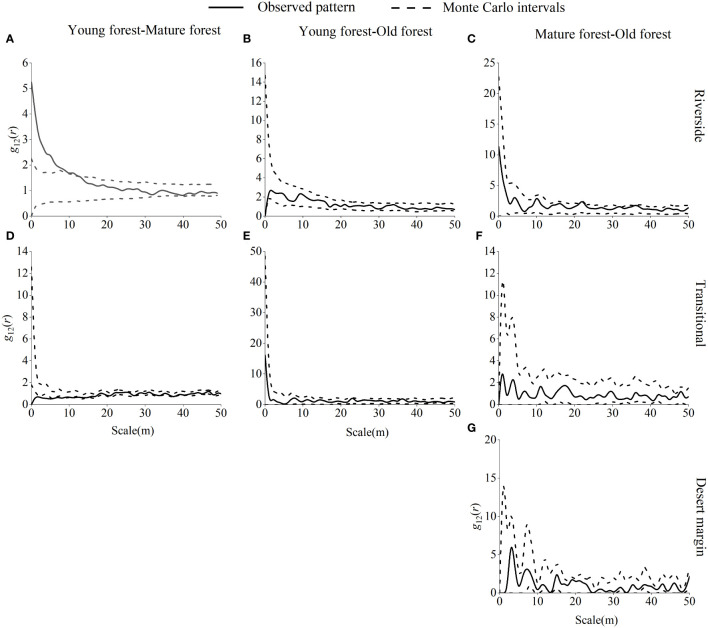
Spatial correlations between *P. euphratica* of different age forests in the riverside **(A–C)**, transitional **(D-F)**, and desert margin habitats **(G)**.

Young and mature *P. euphratica* forests were largely independent of one another, with little spatial association in the riverside habitat and a strong positive correlation at the 0–9 m scale (**Figure 8**). In the transitional habitat, *P. euphratica* forests were mainly independent of each other with no spatial correlation and were significantly negatively correlated at 0–1 m, 3–5 m, 7–8 m, 10 m, 12 m, 15 m, 41 m, and 45 m scales. Both young and old *P. euphratica* forests showed independent relationships with each other and no spatial correlation in the riverside or transitional habitats. The *P. euphratica* mature and old forests were independent of each other, with no spatial correlation in the riverside, transitional, or desert margin habitats.

**Figure 9:**
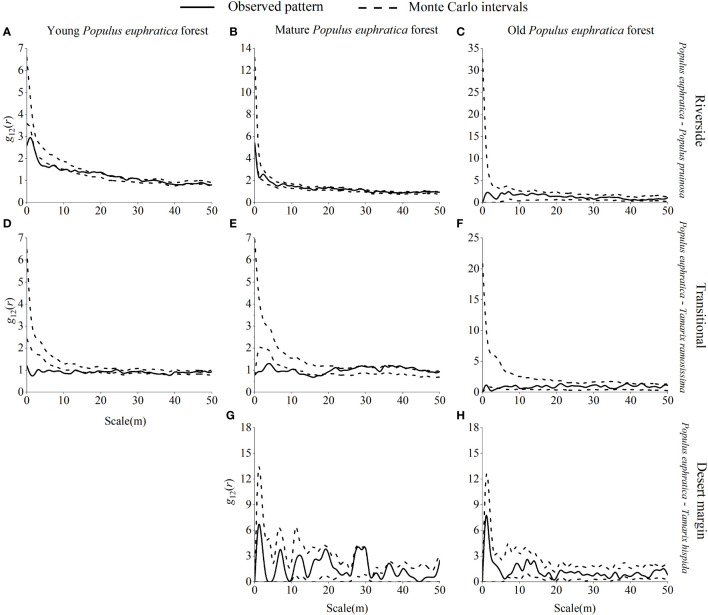
Spatial correlations of *P. euphratica* with *P. pruinosa*, *T. ramosissima* and *T. hispida* in different age forests in the riverside **(A–C)**, transitional **(D-F)**, and desert margin habitats **(G, H)**.

Young *P. euphratica* and *P. pruinosa* were mainly independent of each other in the riverside habitat, with no spatial correlation, and were significantly negatively correlated at 0–1 m, 3–10 m, and 12 m scales and were significantly positively correlated at 21–23 m, 25–26 m, 28 m, and 30 m scales (**Figure 9**). In the transitional habitat, young *P. euphratica* and *T. ramosissima* were primarily independent of one another, with no spatial association and only substantial negative correlations at 0–12 m, 34 m, and 36 m scales. Mature *P. euphratica* and *P. pruinosa* were mainly independent of each other in the riverside habitat, with no spatial correlation, and were only negatively correlated at the 1 m scale. There was a significant negative correlation between mature *P. euphratica* and *T. ramosissima* at 0–18 m in the transitional habitat and a significant positive correlation at 26 m, 29 m, 31–32 m, 35 m, 37–39 m, and 41–46 m scales. Mature *P. euphratica* and *T. hispida* were mainly independent of each other in the desert margin habitat, with no spatial association. Old *P. euphratica* forest and *T. ramosissima* were mainly independent of each other in the riverside habitat and were not spatially related, while old *P. euphratica* forest and *T. ramosissima* and *T. hispida* were mainly independent of each other in the transitional and desert margin habitats and were not spatially related.

### 3.4 Renewal of *P. euphratica* seedlings

**Figure 10:**
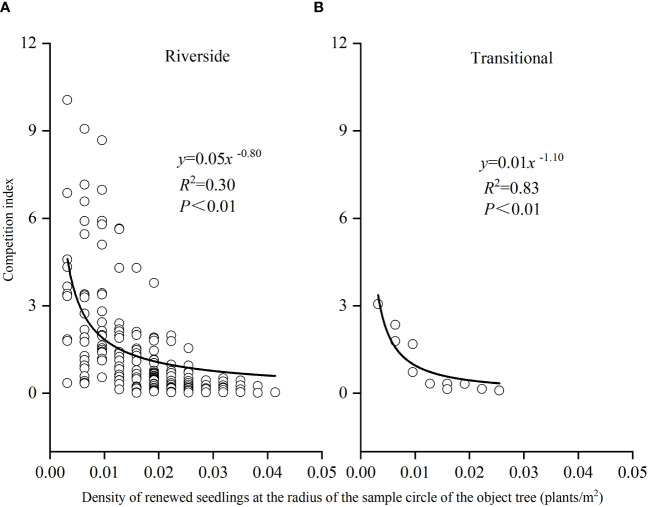
Seedling density and competition intensity of *P. euphratica* object trees renewal in the riverside **(A)** and transitional habitats **(B)**.

The relationship between *P. euphratica* seedling density and the competitive intensity of object tree renewal in both the riverside and transitional habitats obeyed a power function, with *R* ^2^ values of 0.03 and 0.83, respectively (**Figure 10**), and the higher the density of object tree renewal was, the lower the competitive pressure the object tree was subjected to. The number of seedlings at the desert margin was too small to be analyzed.

Correlation analysis between *P. euphratica* seedling density and CI in the riverside, transitional, and desert margin habitats was conducted (**Table 4**). Within a radius of 0–5 m, the CI of *P. euphratica* was positively correlated with surviving seedling density in the riverside and transitional habitats (*P*< 0.01), and the CI was not correlated with the density of all seedlings. Within a 0–10 m radius, the CI of *P. euphratica* was significantly positively correlated with the density of surviving seedlings in the riverside and transitional habitats (*P*< 0.01), and the CI was also significantly positively correlated with the density of all seedlings (*P*< 0.01).

**Table 4:** Correlation between density of renewed seedlings and competition indices.

Sample circle radius range	0-5 m			0-10 m		
Habitat	Riverside	Transitional	Desert margin	Riverside	Transitional	Desert margin
Competition index and all renewal seedling densities	-0.280	-0.463	–	-0.512^**^	-0.575^**^	–
Competition index and density of surviving renewal seedlings	-0.522^**^	-0.793^**^	–	-0.505^**^	-0.858^**^	–

## 4 Discussion and conclusions

### 4.1 Intraspecific and interspecific competition in *P. euphratica*

Competition for resources and space is the main expression of the interaction between plants above- and below-ground. Determining the size of the competitive range is a prerequisite for studying competition among individual plants, and only a true understanding of the competitive range and ecological effects can ensure accuracy in research (Chen et al., 2014). A strategy of gradually increasing the competitive range was employed in this study to determine a reasonable neighbor competition range centered on an object tree, and we found that 10 m was the ideal neighbor range for *P. euphratica* populations. This range may be related to the root system of *P. euphratica*, which is strong, with well-developed lateral roots that extend throughout the soil layer at a depth of 1 m (Si et al., 2007). Moreover, *P. euphratica* roots can penetrate the sand layer up to 10 m below the surface and can continuously draw water and nutrients from the groundwater (Xu, 2022), which leads to an increase in the spacing in *P. euphratica* populations. Long et al. (2021) reported that the J-C effect may also lead to the absence of the same species within a certain distance in a *P. euphratica* community, resulting in increased distances between trees. The decline of *P. euphratica* populations and the death of individuals due to natural factors, such as extreme climatic events and regional water stress (decreasing groundwater depths), can also increase tree spacing (He et al., 2023).

Competitive relationships are prevalent within plant communities, and they affect individual plant growth and development, population structure and dynamics, and community construction. According to the competitive exclusion hypothesis, plant species with the same or similar ecological niches cannot coexist stably, and more competitive species will exclude or eliminate less competitive species (Gause, 1934). In different habitats, *P. euphratica* competition intensity and species diversity differed significantly. Both intraspecific and interspecific competition intensity and species diversity were as follows: riverside habitat > transitional habitat > desert margin habitat. This may be related to the bioecological characteristics of *P. euphratica*. As an important barrier in the Tarim Basin ecosystem, *P. euphratica* is drought-tolerant, salt-tolerant, water-loving, and light-loving. The survival of *P. euphratica* depends on groundwater and on flooding after the annual melting of ice and snow (He, 2022). *Populus euphratica* can access subterranean and riverside water resources in the Tarim River Basin, which greatly enhances its survival, resulting in larger populations of *P. euphratica* in the riverside habitat than in the transitional or desert margin habitats. The density of *P. euphratica* is greater in the riverside habitat, and the population has similar ecological requirements, which leads to competition for resources. Conditions in transitional and desert margin habitats are harsh relative to those in the riverside habitat. In extremely arid climates, strong evaporation from woodland soil causes surface salt accumulation, and a soil environment with high salt and low water potential inhibits the establishment, growth, and development of *P. euphratica* (Kang, 2021), resulting in a decrease in the number and competitive intensity of *P. euphratica* individuals in transitional and desert margin habitats. Moreover, this study showed that the intensity of intraspecific *P. euphratica* competition in the three habitats was much greater than the intensity of interspecific competition, which was a prerequisite for *P. euphratica* populations to coexist stably in the three habitats (otherwise competitive exclusion occurred) (Case, 1999). Forests of *P. euphratica* along the Tarim River are subject to prolonged and extensive periodic flooding (Ma J. L. et al., 2023), which can inhibit the growth of dominant understory plants and thus increase species richness and diversity, consistent with the moderate disturbance hypothesis (Connell, 1978). The competition intensity for young *P. euphratica* forest in the riverside habitat, mature forest in the transitional habitat, and old forest in the desert margin habitat increased. Close to the river, the groundwater depth is shallow, the horizontal root system of *P. euphratica* is well developed, and there is no obvious taproot. Compared with young *P. euphratica* forests in the transitional and desert margin habitats, the development of young *P. euphratica* was better in the riverside habitat. As the distance from the river increases, the groundwater depth increases, horizontal roots decrease, and vertical and downward coarse roots increase, leading to the formation of more obvious taproots (Jing, 2014). Therefore, old *P. euphratica* forest dominates the desert edge. The same tree species have similar ecological requirements, competition is particularly fierce, and competitive pressure is determined by population density restrictions and size differences among individuals (He et al., 2023). The decrease soil water, salt, and nutrients in transitional and desert margin habitats may have been responsible for a decrease in the proportion of young stands and an increase in the stand diameter class structure in the *P. euphratica* population. As *P. euphratica* individuals died, the stand density gradually decreased, and the number of competing trees decreased (Du et al., 2019), which led to a decrease in the competition intensity of the population in the sample plot and a decrease in the CI in the young *P. euphratica* forest.

Among the companion species of *P. euphratica*, the greatest intensity of competition in the riverside habitat was with *P. pruinosa*. In the transitional and desert margin habitats, *P. pruinosa* was reduced in number and did not compete with *P. euphratica*. Consequently, because resources are limited, the overlap of ecological niches between *P. euphratica* and *P. pruinosa* may trigger competition leading to mutual damage, while other species “hitchhike” and coexist, in line with the negative density-dependent (NDD) hypothesis (Zhu Y. et al., 2009; Harms et al., 2000). In addition, in harsh environments where resources are limited, such as the transitional and desert margin habitats, similar species have similar resource needs and competition for limited resources may lead to mutual exclusion (Liu, 2021). Competitive exclusion prevents species with similar ecological niches from coexisting and eventually leads to differences in the development of species within the community and to increasing divergence between species (Slingsby and Verboom, 2006). For example, the response of *P. pruinosa* to the spatial heterogeneity of different habitats reflects its hydrophilic nature and high water requirements. As a typical mesophytic tree species rather than a xerophytic plant, and an area with shallow groundwater and rich organic matter is a suitable habitat for *P. pruinosa* (Han et al., 2013). On the other hand, *Tamarix* species have higher tolerance in both the seedling stage and mature stage, and their roots can absorb water from unsaturated soil (Sher and Marshall, 2003; Sun et al., 2016). These findings show that *P. pruinosa* germination and survival were inhibited by the severe climate in the Tarim River Basin’s transitional and desert margin habitats (Yu et al., 2011). Conversely, *S. ruthenica* and *H. glomeratus* grow in desert margin habitats, and surveys have shown that these two plants grow near *P. euphratica*, which has a caretaker effect; i.e., *P. euphratica* provides a microhabitat for the growth of these two herbs (Zeng et al., 2019).

The Hegyi single-tree CI is commonly used to measure the intensity of competition among plant individuals and can indirectly reflect the allocation of resources by plant individuals. The size of individual trees has a great influence on the strength of competition. By fitting a relationship between DBH and the CI, we found that competition intensity and DBH fit a power function relationship; the CI of the object trees decreased as DBH increased. This finding was consistent with the results of previous studies (Kwong, 2019). When the DBH of *P. euphratica* was less than 30 cm, competition intensity changed significantly with DBH. An object tree’s level of competition was more likely to remain steady when the DBH was greater than 30 cm.

### 4.2 Spatial patterns of *P. euphratica* populations

The interaction between numerous internal and external factors causes the spatial distribution of populations. This pattern is related to the biological characteristics of species and competitive exclusion between populations, but it is also closely related to habitat (including soils, topography, and geomorphology), which is a manifestation of long-term adaptation by species (Wang, 2021). This study showed that young, mature, and old *P. euphratica* forests were randomly distributed in the riverside and transitional habitats and that mature and old forests occurred in the desert margin habitat. The spatial distribution pattern of *P. euphratica* was mainly randomized, suggesting that individuals compete with each other for resources and that intraspecific competition is significantly amplified by the density-constraint effect. As scale increases and intraspecific exclusion is enhanced, populations experience a greater tendency to randomize (Liu, 2021). Stronger self-thinning within *P. euphratica* populations, which limits seedling establishment and growth and reduces population renewal, can also cause a transition from an aggregated to a random distribution (Kang et al., 2019).

Random distributions were also exhibited by *P. pruinosa*, *T. ramosissima*, and *T. hispida*. In the riverside habitat, *P. pruinosa* was partially aggregated at small scales, probably due to the influence of gravity on seed maturation, and the asexual plants produced by root propagation were mostly distributed around the mother plant, leading to an aggregated distribution at young ages. In the process of development from young individuals to mature individuals, self-thinning due to competition for space and nutrients caused the degree of population aggregation to decrease. As the spatial resources required by aging individuals increased, competition became more intense and was coupled with habitat deterioration and a decline in physiological functions. Each year, there were fewer individuals in the aging period, leading to a random distribution (Han et al., 2013). The distribution of *T. ramosissima* was mainly random and clustered in the transitional habitat. Propagation of *T. ramosissima* occurs during the regional flood season, and seeds falling in the transitional habitat germinate in low depressions as the flood recedes, resulting in an aggregated distribution of some young trees (Kang and Han, 2021). However, *T. ramosissima*, and *T. hispida* were more likely to form random distributions because they had numerous small, wind-dispersed seeds and greater dispersal capacity than *P. pruinosa*. Moreover, the salinity of the soil in the transitional habitat and the desert margin made it difficult for many seeds to germinate, and the growth and survival of small trees was difficult. The individuals who survived the harsh environment struggled to develop into medium-sized trees and were randomly distributed. As individuals continued to grow and become more competitive, large trees still exhibited a random distribution (Luo et al., 2021). In contrast, *T. ramosissima* was significantly uniformly distributed at 6 m and 16 m scales in the transitional habitat, indicating that *T. ramosissima* populations experienced strong interindividual interactions at these scales and that the self-sparing effect was stronger to meet their survival needs (Zhang L., 2015).

Interspecific association analysis, an important method for studying interspecific relationships, involves analyzing the spatial distribution of different species, which can reflect mutual attraction or repulsion among species in communities in different habitats; this approach is an important element of the study of speciation and community ecology (Wang et al., 2019). In this study, young, mature, and old *P. euphratica* stands were mainly independent of each other in the riverside habitat, with no spatial correlation. This may be related to different requirements at different growth stages, reflecting the differentiation of environmental resource utilization among young, mature, and old *P. euphratica* individuals, i.e., coexistence within the forest through the utilization of different ecological niches. Young and mature *P. euphratica* forests were significantly negatively correlated at the 0–1 m, 3–5 m, 7–8 m 10 m, 12 m, 15 m, 41 m, and 45 m scales in the transitional habitat, suggesting that to some extent, young and mature *P. euphratica* forests competed for resource utilization. There were significant negative correlations between young *P. euphratica* and *P. pruinosa* at 0–1 m, 3–10 m, and 12 m scales in the riverside habitat, which indicated competitive exclusion between *P. euphratica* and *P. pruinosa* and that both species were strong competitors for resources (Zhao, 2014). Positive correlations between young *P. euphratica* and *P. pruinosa* at 21–23 m, 25–26 m, 28 m, and 30 m scales in the riverside habitat indicated that the two species were similar in their utilization of environmental resources in a small area and jointly resisted the harsh desert environment as well as promoted each other during growth and development (Liu W. L., 2022).

### 4.3 Natural regeneration of *P. euphratica*

Natural regeneration is the main way that plant ecosystems restore themselves (Li et al., 2014), and it is an important process in forest reproduction and sustainable forest management. In this study, the natural regeneration of *P. euphratica* seedlings occurred mainly in the riverside and transitional habitats. There was almost no seedling regeneration at the desert margin, which may be related to the reproductive mode of *P. euphratica*. Initially, *P. euphratica* relied on seed production for population renewal, but due to water scarcity and anthropogenic impacts that limited population renewal by sexual reproduction, most *P. euphratica* reproduction in the Tarim River Basin was clonal reproduction via rootstocks (Gutierrez et al., 2000; Ye et al., 2018). Rootstock regeneration is closely related to soil moisture (Ma J. et al., 2023), and because the desert margin is far from the river and not subject to flooding, the survival and growth of rootstock seedlings is more challenging. Under suitable conditions along the riverside, the lateral roots of *P. euphratica* extend out of the ground and produce new *P. euphratica* seedlings to maintain the population (Li X. et al., 2021). Therefore, when managing *P. euphratica* forests, it is necessary to consider the factors that influence root reproduction and take reasonable measures to promote the growth of *P. euphratica* seedlings. We also discovered that a lower renewal density of *P. euphratica* seedlings experienced greater competitive pressure. Similar results were obtained by Jiang (2022), who studied the effect of the CI on the density of young *Pinus tabuliformis* trees. A greater CI and a smaller DBH led to weaker individual competitive ability, greater competitive pressure from surrounding individuals, and less competitive pressure generated towards the surrounding seedlings; therefore, new seedlings tended to be distributed around individuals with high CIs (Pan et al., 2019).

We found that *P. euphratica* seedling density was higher in the riverside habitat than in the transitional habitat, and there was no regeneration in the desert margin habitat. This may be related to the resource conditions. In the riverside and transitional habitats, species composition is richer, and the number of *P. euphratica* individuals is greater, indicating that the groundwater depth, soil moisture content, salinity, and nutrients in these two habitats are suitable for *P. euphratica* communities. Better conditions lead to greater new seedling density. In the desert margin habitat, species composition is limited, and the number of *P. euphratica* individuals is small, but their DBH is large (Ma et al., 2017), which suggests that groundwater depth in the desert margin habitat is not suitable for a *P. euphratica* community and that seedling regeneration and growth is difficult. There is no replacement by young trees in the desert margin habitat, and with succession, the old trees will die, and the maintenance of a *P. euphratica* forest will be difficult. Moreover, population density has decreased due to intraspecific and interspecific competition and self-thinning in this harsh habitat, which has resulted in increased seedling mortality during development in the desert margin.

## Data availability statement

The raw data supporting the conclusions of this article will be made available by the authors, without undue reservation.

## Author contributions

YXL: Data curation, Investigation, Methodology, Writing – original draft. YZ: Funding acquisition, Investigation, Writing – review & editing. PW: Writing – review & editing. JH: Writing – review & editing. PPL: Writing – review & editing. YJL: Writing – review & editing.

## References

[B1] Blank R. R. (2010). Intraspecific and interspecific pair-wise seedling competition between exotic annual grasses and native perennials: plant–soil relationships. Plant Soil. 326, 331–343. doi: 10.1007/s11104-009-0012-3

[B2] Case T. I. (1999). Illustrated guide to theoretical ecology. Ecology. 80, 2848–2848. doi: 10.1644/1545-1542(2001) 082<0247:>2.0.co;2

[B3] Chai Y. ,. F. (2016). Community assembly during secondary forest succession on the Loess Plateau (China: Northwest University).

[B4] Chen P. Xia J. Ma H. Gao F. Dong M. Xing X.. (2022). Analysis of spatial distribution pattern and its influencing factors of the Tamarix chinensis population on the beach of the muddy coastal zone of Bohai Bay. Ecol. Indicators. 140, 109016. doi: 10.1016/j.ecolind.2022.109016

[B5] Chen Y. Yang J. Zhang P. J. Qing H. Zhao L. Q. Zhang L. (2014). Population structure and spatial point pattern of Helianthemum soon goricum in West Ordos, Inner Mongolia, China. J. Desert Res. 34, 75–82. doi: 10.7522/j.issn.1000-694X.2013.00287

[B6] Connell J. H. (1978). Diversity in tropical rain forests and coral reefs. Science. 199, 1302–1310. doi: 10.1126/science.199.4335.1302 17840770

[B7] Cui Y. H. Han Y. Z. Zhang M. T. Yang X. Q. Zhao Z. H. (2021). Spatial pattern and interspecific association of tree species in coniferous and deciduous broad-leaved mixed forest under different disturbance intensities. J. Appl. Ecology. 32, 2053–2060. doi: 10.13287/j.1001-9332.202106.003 34212611

[B8] Du W. H. Guan W. K. Huo A. D. Yi X. Wei H. (2019). Water and salt characteristics of soil under *populus euphratica* forest in the main stream of tarim river. J. southwest forestry university. 39, 92–99. doi: 10.11929/j.swfu.201812057

[B9] Fraver S. D’Amato A. W. Bradford J. B. Jonsson B. G. Jönsson M. Esseen P. A. (2014). Tree growth and competition in an old-growth Picea abies forest of boreal Sweden: Influence of tree spatial patterning. J. Vegetation Science. 25, 374–385. doi: 10.1111/jvs.12096

[B10] Frymark-Szymkowiak A. Kieliszewska-Rokicka B. (2023). The fine root distribution and morphology of mature white poplar in natural temperate riverside forests under periodically flooded or dry hydrological conditions. Forests 14, 223. doi: 10.3390/f14020223

[B11] Gao H. (2020). Establishment of the forest communtiy and mechanism of regeneration in dominant plant species of sejila mountain in Tibet. [dissertation thesis]. Agricultural and Animal Husbandry college of Tibet University: Linzhi, Tibet.

[B12] Gause G. F. (1934). Experimental analysis of Vito Volterra’s mathematical theory of the struggle for existence. Science. 79, 16–17. doi: 10.1126/science.79.2036.16.b 17821472

[B13] Gou X. X. Ye M. Gao S. F. Xu Q. (2017). Response of radial growth of populus euphratica to climate change in the middle reaches of the tarim river. Acta Bot. Boreal.-Occient. Sin. 37, 1864–1871. doi: 10.7606/j.issn.1000-4025.2017.09.1864

[B14] Gutierrez J. R. Arancio G. Jaksic F. M. (2000). Variation in vegetation andseed bank in a Chilean semi- arid community affected by ENSO1997. J. Vegetation Science. 11, 641–648. doi: 10.2307/3236571

[B15] Han L. Wang H. (2024). Species diversity distribution pattem of desert riparan forest along a groundwater depth gradient in the Tarm Basin. Acta Eoologica Sinica. 44, 832–843. doi: 10.20103/j.stxb.202204281181

[B16] Han L. Xi L. Q. Wang J. Q. Wang H. Z. Yu Z. R. (2013). Life history charctersties and spatial disribution of Populus prninosa population at the upper reaches of Tarim River. Acta Ecologica Sinica. 33, 6181–6190. doi: 10.5846/stxb201305211136

[B17] Hardin G. (1960). The competitive exclusion principle. Science. 131, 1292–1297. doi: 10.1126/science.131.3409.1292 14399717

[B18] Harms K. E. Wright S. J. Calderón O. Hernandez A. Herre E. A. (2000). Pervasive density-dependent recruitment enhances seedling diversity in a tropical forest. Nature. 404, 493−495. doi: 10.1038/35006630 10761916

[B19] Harpole W. S. Tilman D. (2006). Non-neutral patterns of species abundance in grassland communities. Ecol. Letters. 9, 15–23. doi: 10.1111/j.1461-0248.2005.00836.x 16958864

[B20] He X. X. ÜMÜT H. L. Dong Z. W. ASADILLA Y. S. ALSHIR K. R. (2023). Spatial distribution pattern and intraspecific competition of Populs euphratica riparian forests under different water gradients. Acta Ecologica Sinica. 43, 1–10. doi: 10.20103/j.stxb.202205291510

[B21] He Y. X. (2022). Effects of flood irrigation on Populus euphratica in the middleand lower reaches of the Tarim River. [master’s thesis]. Xinjiang Normal University: Urumqi, Xinjiang.

[B22] Hegyi F. (1974). “A simulation model for managing jack-pine stangs,” in Growth models for tree and stand simulation. Ed. Fries J. (Royal College of Forestry, Stockholm, Sweden), 74−90.

[B23] Jiang L. N. (2022). Study on the effects of microhabitat factors on natural regeneration of Pinus tabuliformis plantations on the Loess Plateau. [master’s thesis]. Northwest A&F University: Xianyang, Xian.

[B24] Jing J. L. (2014). Research on the root system distribution and architecture of populus euphratica in the extremely arid region (Haidian (Beijing: Beijing Forestry University).

[B25] Kang J. P. (2021). Study on the spatial pattern dynamics and construction assembly of Populus euphratica communities in the upper reaches of Tarim River. [master’s thesis]. Tarim University: Aral, Xinjiang.

[B26] Kang J. P. Han L. (2021). Spatial distribution and association between Populus pruinosa and Tamarix ramosissima population of desert riparian forest along Tarim River mainstream. J. Cent. South Univ. Forestry Technology. 41, 123–132. doi: 10.14067/j.cnki.1673-923x.2021.02.015

[B27] Kang J. P. Ma Y. Y. Ma S. Q. Xue Z. W. Yang L. L. Han L.. (2019). Dynamic changes of spatial pattern and structure of the Tamarix ramosissima population at the desert-oasis ecotone of the Tarim Basin. Acta Ecologica Sinica. 39, 265–276. doi: 10.5846/stxb201711262112

[B28] Keddy P. A. (1992). Assembly and response rules: two goals for predictive community ecology. J. vegetation science. 3, 157–164. doi: 10.2307/3235676

[B29] Khaine I. Woo S. Y. Kwak M. Lee S. H. Je S. M. You H.. (2018). Factors affecting natural regeneration of tropical forests across a precipitation gradient in Myanmar. Forests. 9, 143–160. doi: 10.3390/f9030143

[B30] Kwong S. (2019). Stand structure and response of growth to climate of Mongolian pine natural forest. [dissertation thesis]. [Haidian (Beijing)]: Beijing Forestry University.

[B31] Lei S. Q. Wang W. J. Wang Y. C. Chen L. J. Wu X. L. Deng Z. W.. (2020). Effects of tamarx ramosisima seedlings on populus euphratica seedlings under various water and salinity conditions. Acta Ecologica Sinica. 40, 7638–7647. doi: 10.5846/stxb201907271586

[B32] Li D. Z. Liu K. Y. Zang R. G. Wang X. P. Sheng L. J. Zhu Z. L.. (2006). Development of the modern niche theory and its main representative genres. Scientia Silvae Sinicae. 43, 88–94. doi: 10.11707/j.1001-7488.20060815

[B33] Li J. Gao X. Xu G. Zheng X. X. (2014). Structure analysis and evaluation of seedlings in evergreen broad-leaved forest in Jiangle Forest Farm, Fujian. J. Northwest A F University-Natural Sci. Edition. 42, 62–68. doi: 10.13207/i.cnki.inwafu.2014.05.002

[B34] Li J. Yu B. Zhao C. Nowak R. S. Zhao Z. Sheng Y. (2013). Physiological and morphological responses of Tamarix ramosissima and Populus euphratica to altered groundwater availability. Tree Physiology. 33, 57–68. doi: 10.1093/treephys/tps120 23243028

[B35] Li M. Y. Deng M. J. Ling H. B. Wang G. Y. Xu S. W. (2021). Evaluation of ecological water security and analysis of driving factors in the lower Tarim River, China Vol. 38 (Arid Zone Research), 39–47. doi: 10.13866/j.azr.2021.01.05

[B36] Li X. Chen X. X. Zhai J. T. Li Z. J. (2021). Differences in clonal growth and photosynthetic physiology between Populus euphratica Oliv. and Populus pruinosa Schrenk. Chin. J. Ecol. 40, 1997–2004. doi: 10.13292/j.1000-4890.202107.019

[B37] Li X. Hou P. Dong X. G. Sun W. (2003). Investigation and analysis on the population density of populus euphratica in zero flow lower reaches of tarim river. J. Xinjiang Agric. University. 26, 41–44.

[B38] Li X. Liu W. S. Zhou W. Chen F. Y. Mu L. Q. (2020). Analysis on community structure and dominant population point pattern of secondary forest of quercus mongolica. Bull. Botanical Res. 40, 830–838. doi: 10.7525/j.issn.1673-5102.2020.06.005

[B39] Liu J. Long A. H. Li J. Yu J. W. Zhang J. (2019). Analysis on runoff evolution laws and trends of three source-streams of Tarim River in recent 60 years. Water Resour. Hydropower Engineering. 50, 10–17. doi: 10.13928/j.enki.wrahe.2019.12.002

[B40] Liu K. Huang B. L. Chen K. Yang M. (2018). Relationship between seedling regeneration, growth and environmental factors in parashorea chinensis natural forest. J. Natural Sci. Hunan Normal University. 41, 47–53. doi: 10.7612/j.issu.2096-5281.2018.04.007

[B41] Liu W. L. (2022). Community characteristics and population distribution pattern of Agropyron mongolicum in desert steppe in heterogeneous habitats. [master’s thesis]. Ningxia University: Yinchuan, Ningxia.

[B42] Liu Y. (2022). Study on community structure and interspecific association of naturalregeneration of three plantations in Beijing Xiaoxishan. [master’s thesis]. Beijing Forestry University: Haidian, Beijing.

[B43] Liu Z. Q. (2021). Maintenance mechanisms of species diversity at different spatial scales in Gaole Mountain National Nature Reserve. Henan Agricultural University: Zhengzhou, Henan.

[B44] Liu Z. J. Hu M. F. Xie Y. Zhou Z. P. Zhang J. D. (2023). Spatial distribution pattern of dominant tree species in Nanling Nature Reserve. J. Sichuan Forestry Sci. Technology. 44, 98–103. doi: 10.12172/202203120001

[B45] Long S. S. Zeng S. Q. Liu F. L. Wang G. X. (2020). Influence of slope, aspect and competition index on the height-diameter relationship of Cyclobalanopsis glauca trees for improving prediction of height in mixed forests. Silva Fennica. 54, 10242. doi: 10.14214/sf.10242

[B46] Long Y. X. Yang X. Cao Y. Lv G. H. Li Y. Pan Y. J.. (2021). Relationship between soil fungi and seedling density in the vicinity of adult conspecifics in an arid desert forest. Forests 12 (1), 92. doi: 10.3390/f12010092

[B47] Luo Y. McIntire E. J. Boisvenue C. Nikiema P. P. Chen H. Y. (2020). Climatic change only stimulated growth for trees under weak competition in central boreal forests. J. Ecology. 108, 36−46. doi: 10.1111/1365-2745.13228

[B48] Luo Y. Zhao Z. X. Huang Z. K. Guan W. K. Huo A. D.. (2021). Spatial distribution pattern and interspecific association of Populus euphratica and Tamarix ramosissima populations in the middle and lower reaches of Tarim River. J. Northeast Forestry University 49, 45–50. doi: 10.13759/j.cnki.dlxb.2021.11.009

[B49] Ma J. M. Ma J. P. Man D. Q. Guo C. X. Zhang Y. N. Zhao P.. (2023). Distribution and regeneration characteristics of natural Populus euphratica forests in Hexi Corridor and their relationship with soil factors. Arid Zone Res. 40, 224–234. doi: 10.13866/j.azr.2023.02.07

[B50] Ma J. L. Shi J. H. Wang X. Y. Baidourela A. Liu M. X. ABULA A. (2023). Effects of flood overflow on soil organic carbon and active components of Populus euphratica forest in the middle reaches of the Tarim River. Arid Zone Res. 40, 1248–1257. doi: 10.13866/j.azr.2023.08.05

[B51] Ma S. Q. Xue Z. W. Yang L. L. Kang J. P. Ma Y. Y. Han L. (2017). Comparison of species diversity under heterogeneous habitats, determination and evaluation of diversity indices of desert riparian forest community in Tarim Basin. Bull. Botanical Res. 37, 961–969. doi: 10.7525/i.issn.1673-5102.2017.06.021

[B52] May R. M. (1973). On relationships among various types of population models. Am. Naturalist. 107, 46–57. doi: 10.1086/282816

[B53] Miao N. Jiao P. Tao W. Li M. Li Z. Hu B.. (2020). Structural dynamics of Populus euphratica forests in different stages in the upper reaches of the Tarim River in China. Sci. Rep. 10, 3196. doi: 10.1038/s41598-020-60139-7 32081960 PMC7035331

[B54] Miao S. H. (2017). Study on the stand spatial structure and species competitionrelationship of natural Machilus versicolora Community in MangdangShan Nature Reserve. [master’s thesis]. Fujian Agriculture and Forestry University: Fuzhou, Fujian.

[B55] Mores G. J. Spadeto C. Dias P. B. dos Santos A. R. Kunz S. H. (2020). Natural regeneration of woody and herbaceous species in ecological restoration areas in the Atlantic Forest.*Revista Brasileira de Ciências Agrárias* . Braz. J. Agric. Sci. 15, 1–10. doi: 10.5039/agraria.v15i4a8160

[B56] Muller-Landau H. C. Wright S. J. Calderón O. Condit R. Hubbell S. P. (2008). Interspecific variation in primary seed dispersal in a tropical forest. J. Ecology. 96, 653–667. doi: 10.1111/j.1365-2745.2008.01399.x

[B57] Niu K. C. Liu Y. N. Shen Z. H. He F. L. Fang J. Y. (2009). Community assembly: the relative importance of neutral theory and niche theory. Biodiversity Science. 17, 579–593. doi: 10.3724/SPJ.1003.2009.09142

[B58] Omelko A. Ukhvatkina O. Zhmerenetsky A. Sibirina L. Petrenko T. Bobrovsky M. (2018). From young to adult trees: how spatial patterns of plants with different life strategies change during age development in an old-growth Korean pine-broadleaved forest. For. Ecol. Manage. 411, 46–66. doi: 10.1016/j.foreco.2018.01.023

[B59] Pan L. Kwong S. Liu Y. Zhang X. Yang X. Shan D. (2019). Tree competition, spatial pattern, and regeneration of a Mongolian pine natural forest in the southern geographical edge. Acta Ecologica Sinica. 39, 3687–3699. doi: 10.5846/stxb201804270955

[B60] Peng W. J. Wang X. M. (2016). Concept and connotation development of niche and its ecological orientation. Chin. J. Appl. Ecology. 27, 327–334. doi: 10.13287/j.1001-9332.201601.005 27228625

[B61] Ripley B. D. (1977). Modelling spatial patterns. Journal of the Royal Statistical Society. Ser. B (Methodological) 39, 172–212. doi: 10.1111/j.2517-6161.1977.tb01615.x

[B62] Sharma L. N. Shrestha K. B. Maren I. E. (2018). Tree regeneration in gap-understory mosaics in a subtropical Shorea robusta (Sal) forest. J. Forestry Res. 30, 2061–2068. doi: 10.1007/s11676-018-0747-x

[B63] Shen C. Nelson A. S. (2018). Natural conifer regeneration patterns in temperate forests across the Inland Northwest, USA. Ann. For. Science. 75, 54–70. doi: 10.1007/s13595-018-0724-8

[B64] Sher A. A. Marshall D. L. (2003). Seedling competition betyen native Populus deltoides ( Salicaceae) and exotic amarixt ramosistma ( lamanicaceaeacross water regimes and substrate types. Am. J. Botany. 90, 413–422. doi: 10.3732/ajb.90.3.413 21659134

[B65] Shu L. Liu Z. G. Dong L. B. (2019). Spatial pattern and regeneration characteristics of main woody species in natural secondary forest in Maoershan, Northeast China. Chin. J. Appl. Ecology. 30, 1945–1955. doi: 10.13287/j.1001-9332.201906.026 31257767

[B66] Si J. H. Feng Q. Li J. L. Zhao J. (2007). Spatial distribution pattern pf Populus euphratica fine roots in desert riparian forest. Chin. J. Ecology. 26, 1–4.

[B67] Slingsby J. A. Verboom G. A. (2006). Phylogenetic relatedness limits co-occurrence at fine spatial scales: evidence from the schoenoid sedges (Cyperaceae: Schoeneae) of the Cape Floristic Region, South Africa. Am. Naturalist. 168, 14–27. doi: 10.1086/505158 16874612

[B68] Sun L. K. Liu W. Q. Chen T. Liu G. X. (2016). Review on mechanism of habitat adaptability and resource value of Tamarix species. J. Desert Res. 36, 349–356. doi: 10.7522/i.issn.1000-694X.2014.00213

[B69] Sun W. W. Chen D. S. Li Z. Y. Li S. Q. Cheng S. Y. Niu X. M.. (2024). Monitoring wetland plant diversity from space: Progress and perspective. Int. J. Appl. Earth Observation Geoinformation. 130, 103943. doi: 10.1016/j.jag.2024.103943

[B70] Sun Z. Wang Y. Pan L. Sun Y. (2022). Hegyi competition index decomposition to improve estimation accuracy of Larix olgensis crown radius. Ecol. Indicators. 143, 109322. doi: 10.1016/j.ecolind.2022.109322

[B71] Svenning J. C. Fabbro T. Wright S. J. (2008). Seedling interactions in a tropical forest in Panama. Oecologia. 155, 143–150. doi: 10.1007/s00442-007-0884-y 17965886

[B72] Vandermeer J. H. (1972). Niche theory. Annu. Rev. Ecol. Systematics. 3, 107–132. doi: 10.1146/annurev.es.03.110172.000543

[B73] Velázquez E. Martínez I. Getzin S. Moloney K. A. Wiegand T. (2016). An evaluation of the state of spatial point pattern analysis in ecology. Ecography. 39, 1042−1055. doi: 10.1111/ecog.01579

[B74] Wand N. Hao Q. Y. (2012). Research progress on influence factors of forest natural regeneration. Guangdong Agric. Science. 39, 67–70. doi: 10.16768/j.issn.1004-874x.2012.06.065

[B75] Wang F. Xiong Z. Dai X. Li Y. Wang L. (2020). The response of the species diversity pattern of Populus to climate change in China. Phys. Chem. Earth Parts A/B/C 116, 102858. doi: 10.1016/j.pce.2020.102858

[B76] Wang J. (2021). A study on survival pressure and competitiveness of *Pinus* Sect. *Cembra* in China. [dissertation thesis]. Haidian, Beijing: Beijing Forestry University.

[B77] Wang L. Chang J. L. Zhou S. B. Wang X. Y. Zhang J. Q. Yan S. K.. (2019). Species diversity and interspecific association of trees in the Yaoluoping Nation Nature Reserve. Acta Ecologica Sinica. 39, 309–319. doi: 10.5846/stxb201710271926

[B78] Wang X. Y. Xu H. L. Pan C. D. Ling H. B. Yuan K. Y. (2017). Population survival characteristics of Populus euphratica which is rare and endangered in the lower reaches of Tarim River. Acta Bot.Boreal.-Occident. Sin. 37, 2282–2289. doi: 10.7606/j.issn.1000-4025.2017.11.2282

[B79] Wang X. Y. Shi J. H. Lu M. X. Bai L. L. Aiiier A. (2020). Effects of flood overtopping on leaf osmotic adjustment substances and antioxidant enzyme activities of natural Populus euphratica forest in the middle reaches of the Tarim River. Arid Zone Research 37, 1544–1551. doi: 10.13866/j.azr.2020.06.20

[B80] Wang Y. F. Qu M. X. Lan H. Y. Duan W. B. Chen L. X. Chun X.. (2020). Spatial pattern and interspecific correlation of dominant population in Spruce-fir-Korean pine forest. J. Cent. South Univ. Forestry Technology. 40, 49−58. doi: 10.14067/j.cnki.1673-923x.2020.01.006

[B81] Wen X. H. Wang Q. B. Pan H. Wang L. R. Chen Y. He D. J. (2022). Interspecific associations of the main tree populations of the Cryptomeria fortunei community in Tianbaoyan. J. For. Environment. 42, 1−10. doi: 10.13324/j.cnki.jfcf.2022.01.001

[B82] Whittaker R. H. Levin S. A. Root R. B. (1973). Niche, habitat, and ecotope. Am. Naturalist. 107, 321–338. doi: 10.1086/282837

[B83] Wiegand T. Gunatilleke S. Gunatilleke N. Okuda T. (2007). Analyzing the spatial structure of a Sri Lankan tree species with multiple scales of clustering. Ecology 88, 3088–3102. doi: 10.1890/06-1350.1 18229843

[B84] Xiang X. Y. Wu G. L. Duan R. Y. Yan Y. M. Zhang X. P. (2015). intraspecific and interspecific competition of Pimus dabeshamesis. Acta Ecologica Sinica. 35, 389–395. doi: 10.5846/stxb201401130102

[B85] Xu H. L. (2022). The patron saint of the desert—Populus euphratica. Friends Science. 8, 42–43.

[B86] Ye Z. Deng R. Wang Y. Wang J. Li J. Zhang F.. (2018). Branching patterns of clonal root of Populus euphratica and its associations with soil factors. J. Beijing Forestry University. 40, 31–39. doi: 10.13332/i.1000-1522.20170426

[B87] Yu J. Wang H. Z. Chen J. L. Han L. (2011). Spatial pattern of Populus euphratica community of desert riparian forest in Tarim River basin. J. Desert Res. 31, 913–918.

[B88] Yusup A. Halik Ü. Abliz A. Aishan T. Keyimu M. Wei J. (2022). Population structure and spatial distribution pattern of Populus euphratica riparian forest under environmental heterogeneity along the Tarim River, Northwest China. Front. Plant Sci. 13, 844819. doi: 10.3389/fpls.2022.844819 35783956 PMC9244701

[B89] Zeng Y. Zhao C. Y. Li C. J. Zheng J. Q. Lu G. H. Li Y. (2019). Spatial distribution pattern and association of Populus euphratica community in different habitats along the Tarim River. Chin. J. Ecology. 38, 3273–3282. doi: 10.13292/j.1000-4890.201911.022

[B90] Zeng Y. Zhao C. Y. Shi F. Z. Schneider M. Lv G. H. Li Y. (2020). Impact of groundwater depth and soil salinity on riparian plant diversity and distribution in an arid area of china. Sci. Rep. 10, 7272. doi: 10.1038/s41598-020-64045-w 32350302 PMC7190620

[B91] Zhang F. Sun J. W. Sun Y. Zheng J. H. Qiao J. R. Zhao M. L. (2021). Effects of different stocking rates on interspecific relationships among dominant species and their spatial distribution characteristics in the *Stipa breviflora* desert steppe. Acta Prataculturae Sinica. 30, 1–11. doi: 10.11686/cyxb2021027

[B92] Zhang L. (2015). A study on the spatial point pattern and potential distribution of Populus euphratica populations in the oasis of lower reaches of Heihe River. [master’s thesis]. Northwest Normal University: Lanzhou, Gansu.

[B93] Zhang L. Lu C. Li X. Wang L. Zhang X. (2012). Age structure and inter-and intra-species competition of Pteroceltis tatarinowii in Huangcangyu Natural Reserve. J. Shanghai Jiaotong University-Agricultural Science. 30, 34−40. doi: 10.3969/J.ISSN.1671-9964.2012.01.006

[B94] Zhang M. Li T. T. Zhang Q. D. Bi R. C. (2017). Study on the spatial distribution patterns and maintaining mechanisms of dominant trees in Taiyue Mountain, Shanxi. Acta Botanica Boreali-Occidentalia Sinica. 37, 782−789. doi: 10.7606/i.issn.1000-4025.2017.04.0782

[B95] Zhang Y. C. (2015). The study of chinese fir stand visual simulation based on crown competition index. [master’s thesis]. Chinese Academy of Forestry: Haidian, Beijing.

[B96] Zhang Y. Deng X. Huang Y. Li Y. Xiang W. Yan W. (2019). Quantification of individual tree competition index taking Chinese-fir plantations in subtropical low hilly area as an example. Polish J. Ecology. 67, 1−16. doi: 10.3161/15052249PJE2019.67.1.001

[B97] Zhang Z. H. Hu G. Qin C. He Y. Li Y. F. (2016). Intraspecific and interspecific competition of dominant tree Castanopsis fissa in Qingxiushan Scenic Spot, Guangxi, Southern China. J. Cent. South Univ. Forestry Technol. 36, 67–71, 85. doi: 10.14067/j.cnki.1673-923x.2016.01.012

[B98] Zhao L. J. (2014). Structure characteristics and influencing factors of Lithocarpus glaber-Cyclobalanopsis glauca community in subtropical evergreen broad-leaved forest (Changsha: Central South University of Forestry & Technology).

[B99] Zhao X. Y. (2021). Research progress on natural forest regeneration. World J. Forestry. 10, 33–42. doi: 10.12677/WJF.2021.101005

[B100] Zhou M. (2019). Effects of reduced plant diversity on species competition and soil environment. [master’s thesis]. Haidian, Beijing: Beijing Forestry University.

[B101] Zhu Y. Mi X. C. Ma K. P. (2009). A mechanism of plant species coexistence: the negative density-dependent hypothesis. Biodiversity Sci. 17, 594–604. doi: 10.3724/SPJ.1003.2009.09183

[B102] Zhuo W. H. (2022). The Quantity Dynamics and Competitive Relationship of MainDominant Populations of Evergreen Deciduous Broad-leaved mixed Forestin Karst Hills of Guilin Southwest China. [master’s thesis]. Guangxi Normal University: Guilin, Guangxi.

